# Rethinking bioinformatics in liquid–liquid phase separation: data resources, predictive models, and an event-centric perspective

**DOI:** 10.1093/bib/bbag254

**Published:** 2026-05-25

**Authors:** Zi-long Yuan, Bo Wang, Yu-lu Chen, Hao-qi Huang, Bin-hao Li, Ahmed Zahoor, Liping Ren, Mengze Du, Rui-qin Fang, Lin Ning

**Affiliations:** School of Medical Technology and Information Engineering, Zhejiang Chinese Medical University, 548 Binwen Road, Binjiang District, Hangzhou 310053, Zhejiang, China; School of Medical Technology and Information Engineering, Zhejiang Chinese Medical University, 548 Binwen Road, Binjiang District, Hangzhou 310053, Zhejiang, China; School of Life Science and Technology, University of Electronic Science and Technology of China, No. 2006 Xiyuan Avenue, West Hi-Tech Zone, Chengdu 611731, Sichuan, China; School of Life Science and Technology, University of Electronic Science and Technology of China, No. 2006 Xiyuan Avenue, West Hi-Tech Zone, Chengdu 611731, Sichuan, China; School of Medical Technology and Information Engineering, Zhejiang Chinese Medical University, 548 Binwen Road, Binjiang District, Hangzhou 310053, Zhejiang, China; School of Medical Technology and Information Engineering, Zhejiang Chinese Medical University, 548 Binwen Road, Binjiang District, Hangzhou 310053, Zhejiang, China; School of Healthcare and Technology, Chengdu Neusoft University, No. 1 Dongruan Avenue, Qingchengshan Town, Dujiangyan District, Chengdu 611844, Sichuan, China; School of Life Science and Technology, University of Electronic Science and Technology of China, No. 2006 Xiyuan Avenue, West Hi-Tech Zone, Chengdu 611731, Sichuan, China; School of Healthcare and Technology, Chengdu Neusoft University, No. 1 Dongruan Avenue, Qingchengshan Town, Dujiangyan District, Chengdu 611844, Sichuan, China; School of Life Science and Technology, University of Electronic Science and Technology of China, No. 2006 Xiyuan Avenue, West Hi-Tech Zone, Chengdu 611731, Sichuan, China; School of Medical Technology and Information Engineering, Zhejiang Chinese Medical University, 548 Binwen Road, Binjiang District, Hangzhou 310053, Zhejiang, China; School of Life Science and Technology, University of Electronic Science and Technology of China, No. 2006 Xiyuan Avenue, West Hi-Tech Zone, Chengdu 611731, Sichuan, China; Yangtze Delta Region Institute (Quzhou), University of Electronic Science and Technology of China, No. 1 Chengdian Road, Kecheng District, Quzhou 324003, Zhejiang, China

**Keywords:** liquid–liquid phase separation, bioinformatics, database, predictive model, LLPS events

## Abstract

Liquid–liquid phase separation (LLPS) has emerged as a fundamental mechanism underlying the formation and regulation of membraneless cellular compartments and is increasingly implicated in diverse physiological processes and diseases. Alongside rapid experimental and high-throughput advances, bioinformatics data resources and computational models have expanded substantially, enabling systematic cataloguing of LLPS-associated components and prediction of phase-separation behavior from molecular features. However, the resulting computational landscape remains highly fragmented. In this review, we provide a comprehensive and critical synthesis of bioinformatics resources and predictive modelling approaches for LLPS. We examine and compare major LLPS databases, highlighting differences in evidence types, curation strategies, coverage, and cross-resource inconsistencies that limit integrative analysis. We then survey computational models across core LLPS prediction tasks, encompassing more than 40 representative algorithms and tracing methodological evolution from classical machine learning to deep learning and large language model-based frameworks. By integrating these advances, we identify a fundamental mismatch between molecule-centric data abstractions and the inherently multicomponent, context-dependent organization of LLPS phenomena. We argue that future progress may benefit from event-centric frameworks that explicitly represent molecular assemblies, contextual conditions and observable phase behaviors, thereby providing a coherent foundation for next-generation LLPS datasets and computational models with improved mechanistic interpretability and translational relevance.

## Introduction

Liquid–liquid phase separation (LLPS) has emerged over the past decade as a fundamental physical principle underlying intracellular organization [1–3]. Since the seminal observation by Brangwynne and colleagues [4] in 2009 that P granules in *Caenorhabditis elegans* exhibit liquid-like behavior, phase separation has been recognized as a widespread mechanism by which cells organize biochemical reactions without the need for membrane-bound compartments [5–8]. Through LLPS, biomacromolecules spontaneously demix into dense and dilute phases once local concentrations exceed critical thresholds, giving rise to dynamic assemblies commonly referred to as biomolecular condensates [9, 10].

A growing number of cellular structures—including stress granules, processing bodies, nucleoli, and Cajal bodies—have been shown to form through phase separation [11–13]. These condensates selectively enrich proteins, RNAs and other macromolecules, thereby establishing spatially confined yet highly dynamic microenvironments that regulate gene expression, signal transduction, RNA metabolism, and stress responses [14, 15]. Unlike classical organelles, phase-separated condensates are typically reversible and responsive to environmental cues [16], enabling rapid cellular adaptation. Increasing evidence further links dysregulated phase behavior to pathological conditions such as neurodegenerative diseases, cancer and viral infection, underscoring the biological and clinical relevance of LLPS [17–20]. Importantly, LLPS is often not determined by an isolated biomolecule alone, but emerges from coordinated interactions among multiple components under specific physicochemical and cellular conditions. In this review, we refer to such context-dependent, multicomponent manifestations of phase separation as LLPS events, a perspective that helps connect the biological organization of condensates with the computational abstractions currently used to describe them.

At the molecular level, proteins involved in LLPS are frequently enriched in low-complexity domains and intrinsically disordered regions (IDRs) [21, 22]. The conformational plasticity and compositional heterogeneity of these regions complicate structure-centric analyses and challenge traditional experimental approaches [23, 24]. While techniques such as fluorescence recovery after photobleaching (FRAP) [25] and *in vitro* droplet reconstitution are widely used assays for validating phase separation, they are inherently low-throughput and labor-intensive [26]. As a consequence, experimental characterization alone is insufficient [27] to explore the rapidly expanding space of candidate molecules, interactions and conditions associated with LLPS [28]. In parallel, advances in high-throughput sequencing [29], proteomics [30], and systems biology [31] have generated vast amounts of molecular data relevant to phase separation [32]. This data-rich environment has catalyzed a shift in LLPS research from predominantly experiment-driven discovery toward data-driven and computationally assisted analysis [33, 34]. Bioinformatics approaches have become indispensable for integrating heterogeneous datasets, identifying potential phase-separating components and developing predictive models of LLPS-related behavior at scale [35–37].

Over the past decade, a diverse ecosystem of LLPS-related databases and computational prediction models has emerged [38, 39]. These resources have enabled systematic screening of proteins and RNAs, characterization of sequence features associated with phase behavior and large-scale comparative analyses across species and conditions [33]. However, the rapid expansion of data and methods has also exposed conceptual limitations [40]. Most existing computational studies implicitly treat LLPS as an intrinsic property of individual biomolecules, abstracting phase separation into molecule-centric prediction tasks rather than representing it as a structured, context-dependent biological event. Such abstractions, while practical, only partially capture the conditional, multi-component, and context-dependent nature of LLPS *in vivo* [41, 42].

In this review, we provide a comprehensive and critical synthesis of bioinformatics applications in LLPS research, focusing on data resources and computational prediction models. We systematically survey major public databases cataloging LLPS-related molecules, condensates and disease associations, and we review more than 40 computational approaches spanning traditional machine learning, deep learning and protein language model-based methods. Beyond summarizing existing efforts, we articulate a unifying event-centric perspective that reframes LLPS as a structured biological event. By aligning data representation and modeling strategies with the multi-component organization of phase separation, we aim to outline future directions for integrative, mechanistically informed and translationally relevant LLPS bioinformatics.

## Landscape and motivation for a comprehensive review of liquid–liquid phase separation computational research

**Figure 1:**
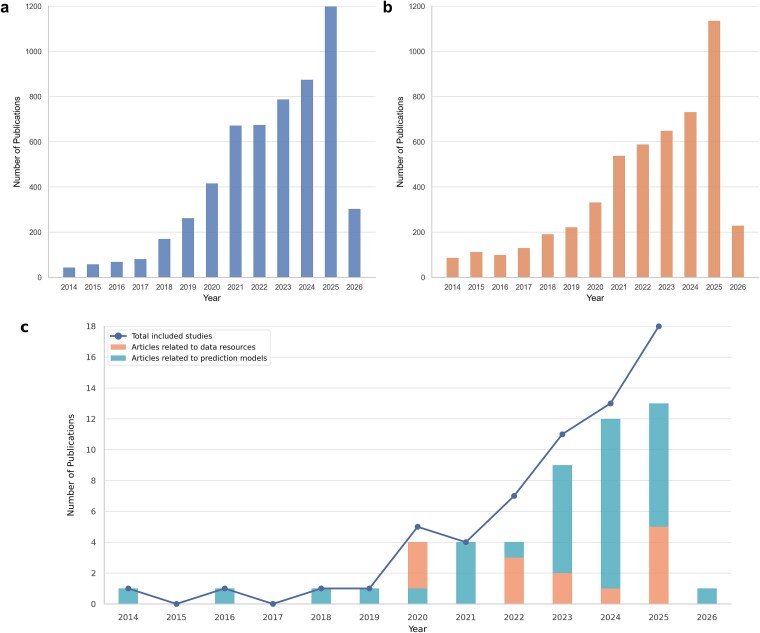
Longitudinal trends in LLPS-related publications and temporal distribution of reviewed literature. Panels a and b present the annual number of LLPS related research articles retrieved from PubMed and Web of Science, respectively, using LLPS-related keywords over the period January 2014–February 2026. Panel c depicts the yearly distribution of the review articles included in this study. The stacked bars represent articles related to data resources and prediction models, whereas the overlaid line (total included studies) indicates the total number of included reviews per year.

Over the past decade, research on LLPS has experienced sustained and rapid growth, driven by continuous experimental discoveries and conceptual advances. Bibliometric analyses based on PubMed and Web of Science reveal a steady increase in LLPS-related publications from January 2014 to February 2026 (Fig. 1a and b), reflecting the consolidation of phase separation as a central paradigm in cell biology, biophysics, and disease research. This expansion also mirrors the growing recognition of biomolecular condensates as dynamic organizational principles that can respond to cellular state and environmental perturbations. Importantly, the development of the field has been both quantitative and conceptual, with LLPS now implicated in an increasingly broad spectrum of cellular processes and physiological contexts.

Early LLPS studies primarily focused on protein- and RNA-driven condensates, emphasizing sequence features such as low complexity and intrinsic disorder. More recent work has substantially broadened this view, demonstrating that additional classes of biomolecules—including glycogen and other polysaccharides—can participate in or modulate phase separation [43–45]. These findings underscore LLPS as a collective and context-dependent phenomenon shaped by multiple interacting components and environmental conditions. As mechanistic understanding becomes increasingly complex, analytical frameworks capable of integrating heterogeneous molecular species and contextual information are required. In parallel, computational and bioinformatics research on LLPS has continued to evolve. Curated databases have been developed to collect experimentally validated phase-separating proteins, RNAs, condensates and disease links, while predictive models have progressed from early physicochemical feature-based approaches to machine learning, deep learning and, more recently, large pretrained protein language models. Together, these developments have enabled large-scale screening and hypothesis generation, substantially accelerating LLPS research.

**Figure 2:**
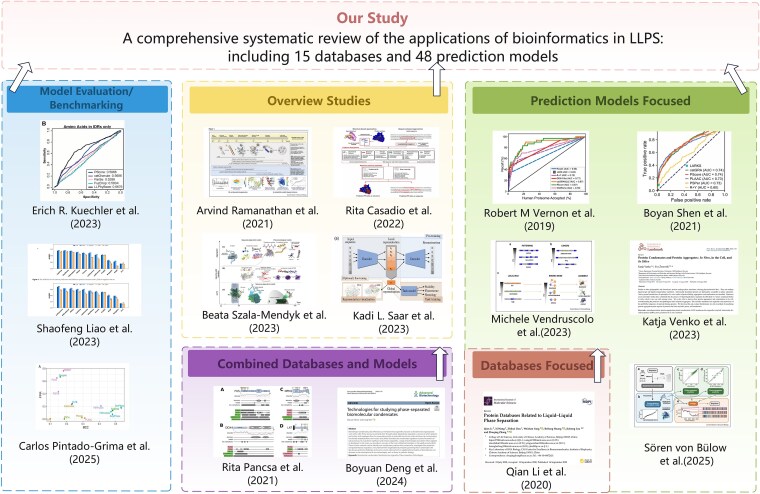
Positioning of this review relative to existing bioinformatics-focused LLPS surveys. This figure compares fifteen previously published reviews on LLPS bioinformatics, grouped according to their primary scope: five focusing exclusively on LLPS prediction models, one dedicated to data resources, two integrating both resources and predictive models, four providing broad descriptive overviews, and three centered on benchmarking or systematic evaluation of LLPS predictors.

To date, approximately 15 review articles have examined bioinformatics and computational aspects of LLPS, encompassing database construction, predictive modelling, and benchmarking efforts (Fig. 2). However, closer inspection reveals several recurring limitations. First, a number of early surveys have become outdated as LLPS datasets and computational tools have expanded rapidly in both scale and experimental modalities [46]. Second, many more recent reviews remain narrowly centered on molecule-centric tasks—most commonly protein-level LLPS propensity prediction—without systematically accounting for partner dependence, environmental factors such as temperature, pH and ionic strength, or multicomponent competition that governs *in vivo* partitioning and context specificity [47–51]. Third, benchmark-oriented studies [40, 52, 53] that apply stringent filtering criteria often yield small datasets, limiting practical utility and rendering existing benchmarks ill-suited to represent the conditional and multicomponent nature of LLPS.

These review-level limitations reflect deeper challenges within the computational landscape itself. LLPS databases differ substantially in curation criteria, evidence types, update cadence and annotation granularity, while extensive cross-resource redundancy complicates harmonization [54–57]. Inconsistent identifier mapping and heterogeneous metadata schemas further undermine interoperability, making it difficult to trace the same molecule, condensate or experiment across resources. On the modelling side, prediction methods vary widely in task formulations, training data and evaluation protocols; even when addressing ostensibly similar questions, studies frequently adopt divergent definitions of positive and negative samples, representations and performance metrics [58–62]. As a consequence, computational outputs are often not directly comparable or readily integrated, and methodological advances do not consistently translate into improved biological interpretability or robust, context-relevant hypotheses [63].

Collectively, these observations highlight a widening gap between the pace of LLPS research and the scope of existing computational syntheses. The absence of a comprehensive and up-to-date review limits researchers’ ability to navigate an increasingly complex ecosystem of data resources and predictive models, leading to duplicated efforts, inconsistent terminology, and uneven adoption of best practices. Moreover, the prevailing molecule-centric perspective constrains how computational problems are formulated and evaluated, frequently overlooking the conditional, multicomponent, and context-dependent organization of LLPS *in vivo*. Motivated by these challenges, the present review aims to systematically organize LLPS-related data resources and computational models, critically assess their assumptions and limitations, and establish a foundation for event-centric and integrative computational frameworks that more faithfully reflect the biological organization of LLPS. The scope of the computational literature examined in this review, including temporal publication trends of LLPS-related data resources and predictive models, is summarized in Fig. 1c, while detailed search strategies, inclusion and exclusion criteria and full query terms are provided in Supplementary Fig. S1 and Supplementary Table S1.

## Liquid–liquid phase separation data resources: scope, curation strategies, and structural limitations

### Overview of liquid–liquid phase separation data resources and organizational paradigms

Over the past decade, a diverse and rapidly expanding collection of public databases has been developed to support data-driven studies of LLPS. These resources reflect both the growing recognition of LLPS as a fundamental biological process and the increasing demand for systematic organization of experimentally derived knowledge. Despite differences in scope and design, most existing LLPS databases can be broadly grouped into three categories based on their primary annotation focus and dominant data presentation form: molecule-centric datasets, condensate- or organelle-oriented resources, and disease-associated LLPS databases; notably, the molecule-centric category can be further subdivided into protein-centric and RNA-related resources. Table 1 provides an overview of representative LLPS-related databases, including their database name, version, resource type, number of entries, associated journal publication, release date, URL, and brief notes. Because some resources contain information relevant to more than one category, classification in this review follows the main organizational unit of each database, while cross-category features are indicated in the Notes column of Table 1. For databases with multiple released versions, the release dates of different versions are listed separately to facilitate comparison and statistical analysis across database updates.

**Table 1:** Overview of LLPS related databases.

**Database**	**Version**	**Type**	**Entry**	**Journal**	**Date**	**URL**	**Notes**
PhaSepDB [64–66]	V3.0	Protein	3484	Nucleic Acids Research	2025/10/8	https://db.phasep.pro/	MLOs
	V2.1	Protein	9492	Nucleic Acids Research	2023/1/6	http://db2.phasep.pro/	MLOs
	V1.0	Protein	2914	Nucleic Acids Research	2020/1/8	/	/
ricePSP [67]	V1.0	Protein	10 654	Genome Biology	2025/11/17	https://ricepsp.github.io/	Proteins in rice
BAV-LLPS [68]	V1.0	Protein	5278	Bioinformatics	2025/10/2	https://bav-llps-db.bioinformatica.org/	Proteins in bacteria, archaea, and viruses
PhaSeDis [69]	V1.0	Disease	931	Genomics, Proteomics & Bioinformatics	2025/5/10	http://mlodis.phasep.pro	LLPS factors and related diseases
RPS [70, 71]	V2.0	RNA	171 301	Nucleic Acids Research	2025/1/6	https://rps.renlab.cn/	/
	V1.0	RNA	42 417	Nucleic Acids Research	2022/1/7	/	/
MLOsMetaDB [38]	V1.0	Protein	12 038	Protein Science	2024/1/1	http://mlos.leloir.org.ar	MLOs
CD-CODE [72]	V1.0	Biomolecular Condensates	9861	Nature Methods	2023/4/6	https://cd-code.org/	/
LLPSDB [73, 74]	V2.0	Protein	2917	Bioinformatics	2022/1/13	http://bio-comp.org.cn/llpsdbv2	Proteins in vitro
	V1.0	Protein	1175	Nucleic Acids Research	2020/1/8	http://bio-comp.org.cn/llpsdb	/
RNAPhaSep [75]	V1.0	RNA	1113	Nucleic Acids Research	2022/1/7	http://www.rnaphasep.cn	/
PhaSePro [76]	V1.0	Protein	121	Nucleic Acids Research	2020/1/8	https://phasepro.elte.hu	Driver proteins in vivo
DrLLPS [77]	V1.0	Protein	437 887	Nucleic Acids Research	2020/1/8	http://llps.biocuckoo.cn/ http://bio-comp.ucas.ac.cn/llpsdb	Proteins in eukaryotes

Protein-centric databases constitute the earliest and most extensively developed class of LLPS resources. Databases such as LLPSDB [73, 74] and PhaSePro [76] curate experimentally validated phase-separating proteins and, in some cases, delineate LLPS-driving regions at the sequence level. These resources typically emphasize low-complexity domains, IDRs and physicochemical features associated with phase behavior, providing high-confidence reference datasets that have been widely used for computational model development. To improve coverage across species, integrative resources such as DrLLPS [77] and BAV-LLPS [68] further expand protein-centric collections through homology-based inference, substantially increasing dataset size while introducing heterogeneous evidence confidence. Complementing protein-focused resources, a smaller but growing set of databases addresses the role of RNA in LLPS, such as RNAPhaSep [75] and RPS [70, 71]. These databases collect information on RNAs that participate in or regulate phase separation, highlighting the importance of protein–RNA interactions in condensate formation. In parallel, condensate- or organelle-oriented databases, exemplified by PhaSepDB [64–66], CD-CODE [72], and MLOsMetaDB [38], organize LLPS-related information at the level of membraneless organelles, linking molecular components to specific cellular structures and subcellular localizations. This higher-level abstraction provides a bridge between molecular annotations and cellular phenotypes. Finally, PhaSeDis [69], the disease-oriented LLPS resources integrate phase separation information with pathological contexts, cataloging associations between LLPS-related molecules, condensates and human diseases. Such databases support translational studies by connecting LLPS dysregulation to disease mechanisms and clinical phenotypes.

Collectively, these databases provide an essential foundation for computational LLPS research by aggregating dispersed experimental evidence into accessible resources. However, despite differences in annotation scope and granularity, the dominant organizational paradigm across all three categories remains molecule-centric. Individual proteins or RNAs typically serve as the primary units of annotation, while cooperative interactions, contextual dependencies, and event-level outcomes are only partially represented. This shared abstraction underlies both the strengths and limitations of current LLPS data resources, highlighting the importance of evidence-aware annotation and curation.

### Evidence hierarchy and curation strategies

The reliability and utility of LLPS data resources are fundamentally shaped by the types of experimental evidence they curate and the strategies used to organize such evidence. Existing LLPS databases integrate information derived from a wide spectrum of experimental approaches, ranging from low-throughput, high-confidence assays to indirect or large-scale experimental observations. However, the heterogeneity of evidence types is often only partially reflected in database annotations, complicating downstream interpretation and computational use. For the purpose of this review, we use “LLPS-related” as a working, evidence-aware term for molecules, condensates or records that are explicitly connected in the source literature to phase-separation phenomena or to closely related condensate contexts, while recognizing substantial differences in evidential strength. Records supported by direct experimental observations of phase behavior or material properties *in vitro* and/or *in vivo* are treated as higher-confidence LLPS evidence. By contrast, punctate localization, colocalization with membraneless organelles, or broader MLO association alone are considered indirect but biologically informative evidence, unless accompanied by additional observations consistent with phase separation. Accordingly, homology-based, text-mined, or predictor-linked records are interpreted here as putative or lower-confidence extensions rather than equivalent substitutes for experimentally validated LLPS evidence.

At one end of the spectrum, classical *in vitro* assays provide the strongest direct evidence for phase separation. These include droplet formation assays under controlled conditions and FRAP, which assess the material properties and dynamics of condensates [78]. Databases such as LLPSDB [73, 74] and PhaSePro [76] rely heavily on manual expert curation of such studies, extracting detailed annotations related to experimental conditions, sequence regions driving phase separation and qualitative phase behavior. While these data offer high confidence and mechanistic insight, they are inherently limited in scale due to the labor-intensive nature of the experiments and the curation process. In contrast, *in vivo* observations—such as the formation of punctate structures in cells or the localization of proteins to membraneless organelles—provide valuable physiological context but often represent indirect evidence of LLPS. These observations may reflect phase separation, but they can also arise from alternative mechanisms, including scaffold-based assembly or stable protein complexes. The extent to which databases distinguish between direct phase separation assays and indirect cellular observations varies substantially, introducing ambiguity in evidence interpretation.

To address scalability, several databases employ hybrid curation strategies that combine manual annotation with homology-based inference or automated text-mining pipelines. Resources such as DrLLPS and BAV-LLPS expand LLPS-associated protein sets by extrapolating experimentally validated cases to homologous proteins, thereby increasing coverage across species [68, 77]. In a related but more prediction-oriented direction, ricePSP extends LLPS resource coverage to rice proteins, but because its entries are derived entirely from computational prediction rather than direct experimental validation, it should be interpreted as a predictor-linked, lower-confidence resource within the present evidence hierarchy [67]. As LLPS-related data have expanded at scale, redundancy and fragmentation across different LLPS databases have become increasingly evident. In response, secondary integration efforts have begun to emerge; e.g. MLOsMetaDB reorganizes and harmonizes data from PhaSePro, LLPSDB, PhaSepDB, and DrLLPS to establish a resource encompassing both LLPS- and MLO-related entries. More recent database updates, exemplified by advanced versions of PhaSepDB, incorporate artificial intelligence-assisted literature mining via agent-based pipelines that automatically extract information on phase-separation processes from the literature, followed by manual cross-validation, to systematically identify candidate LLPS-related entities. While these approaches enhance data volume and update efficiency, they also introduce variability in evidence confidence and annotation granularity.

From the perspective of database iteration, LLPS data resources have undergone a clear methodological transition—from predominantly manual, expert-driven collection to AI-assisted organization—and a parallel rebalancing of content priorities: early releases were often fragmented and partial, subsequent updates pursued broader coverage by incorporating high-throughput MLO-related entries (and, in some resources, predictor-linked annotations), whereas the most recent releases increasingly re-emphasize evidence stratification and the re-curation of experimentally validated phase-separation records to improve reliability and downstream usability. This trajectory is illustrated by PhaSepDB, which initially compiled 2914 non-redundant LLPS-related proteins (v1.0), expanded in v2.1 to include 1419 PS entries plus 8073 MLO entries (9492 total entries), and most recently released v3.0 with 3484 expert-curated PS entries enabled by an LLM-based agentic extraction workflow followed by expert verification [65, 66, 69]. Collectively, current LLPS databases balance competing demands for data quality, coverage, and scalability. However, inconsistent treatment of experimental evidence types and limited standardization of confidence levels pose challenges for computational modeling, underscoring the need for clearer evidence hierarchies and more transparent curation frameworks.

### Redundancy and fragmentation across liquid–liquid phase separation databases

**Figure 3:**
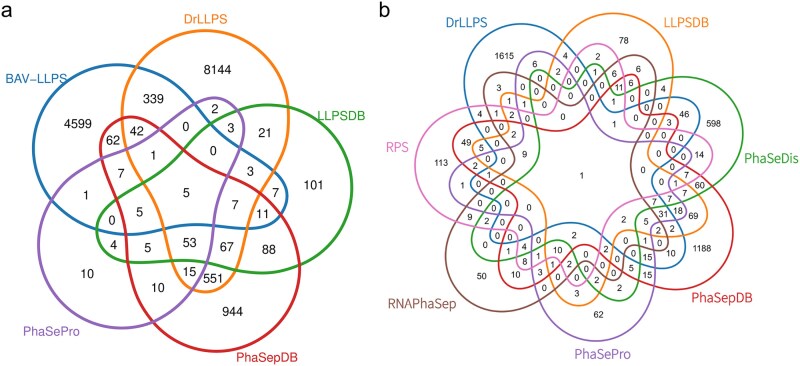
Overlap analysis of phase-separation databases using UniProt-based and PMID-based comparisons. The Venn diagrams illustrate the overlap of LLPS-related resources at two levels using the latest released versions of all databases included in the analysis. Panel a shows the intersections among five phase-separation databases based on UniProt protein identifiers, representing overlap at the protein level. Panel b shows the intersections among nine databases based on PubMed identifiers (PMIDs), reflecting overlap at the literature level; for this analysis, RPS was restricted to entries from Results of reviewed LLPS-associated RNAs (excluding Results of high-throughput LLPS enrichment LLPS-associated RNAs, Results of high-throughput LLPS perturbation LLPS-associated RNAs and Results of predicted LLPS-associated RNAs), and LLPSDB was limited to the Phase_separation_unambiguous dataset within Unambiguous System (excluding entries from ambiguous systems and non-phase-separating datasets). In each panel, the intersecting areas indicate the number of shared entries across the corresponding databases.

The rapid proliferation of LLPS-related databases has resulted in substantial redundancy and fragmentation across resources, posing challenges for data integration and comparative analysis. Cross-database comparisons based on UniProt identifiers (Fig. 3a) reveal that only a limited core set of LLPS-associated proteins is consistently shared among multiple databases, whereas a large fraction of entries appears in only one or a small subset of resources. This pattern reflects divergent curation criteria, evidence thresholds, and expansion strategies rather than fundamental disagreement regarding LLPS biology. Interestingly, analyses at the literature level reveal a contrasting picture. When overlap is assessed using PubMed identifiers (Fig. 3b), many LLPS databases draw upon a largely shared body of foundational studies. However, these studies are abstracted, filtered, and annotated in different ways, resulting in distinct representations of LLPS knowledge. This discrepancy indicates that fragmentation arises less from differences in experimental evidence itself than from differences in how evidence is interpreted and structured within databases.

Such fragmentation has important implications for computational research. Predictive models trained on data from a single database may inadvertently learn database-specific biases, limiting their generalizability across datasets [79, 80]. Inconsistent inclusion criteria across databases further complicate model development: a protein curated as LLPS-associated in one resource may be missing from others or assigned to a different LLPS role, such as a driver or a client, creating divergent training sets. As a result, models trained on resource-specific datasets are harder to compare fairly. Moreover, heterogeneous annotation granularity across databases constrains integrative analysis. Some resources provide residue-level annotations or detailed experimental metadata, whereas others offer only protein-level labels. As a result, constructing unified training or evaluation datasets often requires extensive manual harmonization, introducing additional uncertainty and limiting scalability.

Together, redundancy and fragmentation across LLPS databases highlight a structural challenge that extends beyond data volume. While diversity of resources reflects the dynamic growth of the field, the lack of standardized abstraction and comparability constrains both algorithm development and biological interpretation. In practice, this limits reproducibility across studies, complicates cross-database benchmarking, and increases the risk that models capture database-specific labeling conventions rather than transferable biological signals. More fundamentally, when annotations remain anchored to heterogeneous molecule-level entries with incomplete context and provenance, it becomes difficult to assemble coherent, system-level views of condensates or to trace how evidence supports a given label. These limitations point toward the need for higher-level data representations that can reconcile heterogeneous annotations within a coherent framework.

### Beyond molecule-centric databases: unmet needs for event-level representation

Taken together, the characteristics of current LLPS databases reveal a fundamental structural limitation rooted in molecule-centric abstraction. Across protein-, RNA-, condensate- and disease-oriented resources, individual biomolecules typically serve as the primary units of annotation, while cooperative interactions, contextual dependencies, and collective outcomes are represented only implicitly or not at all. Although this abstraction has been effective for early discovery and large-scale screening, it increasingly constrains the biological fidelity of LLPS data representation. As LLPS research moves toward mechanistic and system-level questions, the lack of explicit links between molecules, conditions, and phenotypic consequences becomes a primary bottleneck for synthesis and reuse.

LLPS can be conceptualized as an event-level phenomenon that arises from coordinated interactions among multiple molecular components under specific physicochemical and cellular conditions. Critical dimensions of phase separation events—including component composition, interaction stoichiometry, environmental context, and functional outcomes—are often fragmented across separate database entries or absent altogether. As a result, biologically distinct LLPS events may be collapsed into identical molecular annotations, obscuring conditional behaviors and limiting interpretability. For example, the same protein may participate as a scaffold, client, or regulator depending on partners and cellular state, yet such role switching is rarely captured by molecule-level records. Existing databases largely capture static molecular and experimental snapshots, whereas LLPS is a dynamic process. Consequently, current datasets generally lack systematic curation and representation of temporal dynamics and process-level changes.

These limitations may have important implications for computational modeling. When training data are organized solely around individual molecules, predictive tasks are naturally framed in terms of intrinsic propensities rather than conditional event outcomes. Even highly expressive models are therefore restricted by data abstractions that do not reflect the organization of the underlying biological process. This mismatch helps explain why improvements in predictive accuracy do not always translate into mechanistic insight or robust generalization across contexts. In particular, models may overfit to dataset-specific labeling conventions or proxy features, such as disorder content, that correlate with curation choices but do not capture event-level determinants or mechanistic drivers of phase behavior.

Addressing these challenges requires a shift toward event-centric LLPS data models that explicitly represent multi-component assemblies, contextual variables, and observable outcomes within unified structures. Such representations would enable more faithful integration of heterogeneous evidence and, importantly, reshape how computational problems are formulated. When LLPS is encoded at the level of structured events rather than isolated molecules, predictive modeling naturally shifts toward reasoning about conditional behaviors, interaction dependencies, and context-specific outcomes, creating a more appropriate foundation for advancing LLPS computational analysis. Practically, this also facilitates interoperable benchmarking by enabling consistent definitions of positives/negatives at the event level and supporting cross-database harmonization without discarding contextual detail.

## Computational modelling for liquid–liquid phase separation: tasks, modelling paradigms and emerging directions landscape of liquid–liquid phase separation predictive modelling

Computational modelling has become an indispensable component of LLPS research, enabling systematic analysis and large-scale screening beyond the reach of experimental approaches alone [81, 82]. Over the past decade, a growing number of predictive models have been developed to infer LLPS-related properties from molecular data, reflecting both the increasing availability of curated datasets and advances in machine learning and artificial intelligence [83, 84]. Our survey suggests a rapidly expanding yet uneven methodological landscape, largely shaped by task formulation, data availability, and modelling paradigms.

**Figure 4:**
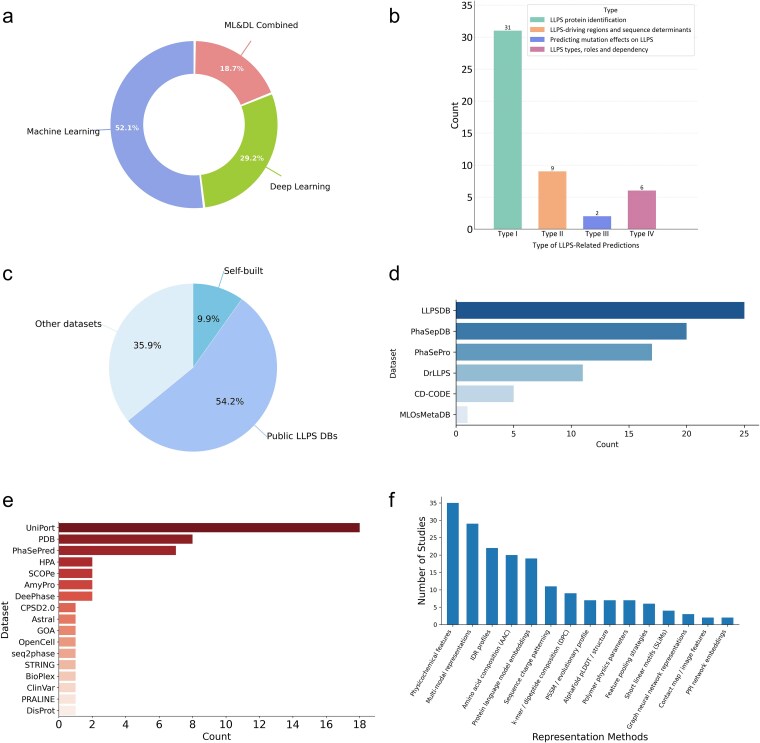
Comprehensive methodological characterization of LLPS protein prediction models with respect to algorithmic paradigms, predictive tasks, dataset utilization, and feature representation strategies. Panels a–f summarize the methodological characteristics of 48 representative LLPS prediction models. Panel a illustrates the proportional distribution of models according to their algorithmic paradigm, classified into machine learning-based, deep learning-based, and hybrid approaches. Panel b categorizes the models based on their primary predictive tasks. Panel c presents the distribution of dataset sources used for model development and evaluation, grouped into three categories: publicly available LLPS databases, self-constructed datasets, and other external resources. Panels d and e further quantify the frequency of dataset utilization, distinguishing between the usage of LLPS-specific datasets and other datasets. Panel f provides a summary of feature representation strategies, showing the number of studies employing each type of feature descriptor.

From a task-oriented perspective, current LLPS prediction models can be broadly grouped into four categories: (i) identification of phase-separating proteins, (ii) prediction of LLPS-driving regions or sequence determinants, (iii) assessment of mutation effects on phase separation behavior, and (iv) classification of LLPS types, roles, or dependency patterns, such as scaffold–client relationships. Protein-level LLPS identification dominates the field, whereas mutation-oriented and type/classification tasks remain comparatively underexplored, likely reflecting both limited high-quality labelled data and increased biological complexity (Table 2, Fig. 4b). Methodologically, the distribution of models across machine learning, deep learning and hybrid paradigms further illustrates how modelling choices have evolved in parallel with dataset growth (Fig. 4a). A complete catalogue of the models included in this review is provided in the Supplementary file. Notably, despite the breadth of protein-centric predictors, dedicated models for LLPS-related RNA entities or RNA-driven phase-separation behavior remain scarce, highlighting an important opportunity to incorporate protein–RNA co-regulation into future predictive frameworks.

**Table 2:** Categorization of LLPS prediction tasks, number of studies, and representative models.

**Task type**	**Number**	**Models**
Task I. LLPS protein identification	31	catGRANULE [85], FLFB [86], LLPhyScore [79], PSPer [87], PSPire [88], MolPhase [89], Pscore [90], PICNIC [91], PSPHunter [92], PSAP [93], PSPredictor [94], FuzDrop [95], GP-GNN [96], Droppler [97], MambaPhase [98], snLLPS [99], PredLLPS_PSSM [100], PSTP [101], Opt_PredLLPS [102], catGRANULE 2.0 ROBOT [103], DeePhase [104], ML_LM [105], Ka Yin Chin *et al.* (BMC Bioinformatics, 2024) [106], Soren von Bulow *et al.* (PNAS,2025) [107], Wesley W. Oliver *et al.* (The Journal of Physical Chemistry B, 2025) [108], Ashwin Lahorkar *et al.* (IEEE/ACM Transactions on Computational Biology and Bioinformatics, 2022) [109], Pratik Mullick *et al.* (Biomolecules, 2022) [110], Qinglan Ma *et al.* (Life (Basel), 2023) [111], Zahoor Ahmed *et al.* (International Journal of Biological Macromolecules, 2024) [112], Jiyan Wang *et al.* (International Journal of Biological Macromolecules, 2023) [113], Wenbin Li *et al.* (Briefings in Bioinformatics, 2025) [114]
Task II. LLPS-driving regions and sequence determinants	9	ParSe [115], dSCOPE [116], ParSe 2.0 [117], PLAAC [118], IFF [119], TIDGN [120], IDR-Puncta ML Model [121], PhaSeMotif [122], Yumeng Zhang *et al.* (Elife, 2025) [123]
Task III. Predicting mutation effects on LLPS	2	PSMutPred [124], PhosLLPS [125]
Task IV. LLPS types, roles, and dependency	6	PULPS [126], PhaSePred [127], Seq2Phase [128], PhaseNet [129], Zahoor Ahmed *et al.* (Proteomics, 2024) [130], Zahoor Ahmed *et al.* (Briefings in Bioinformatics, 2025) [131]

Notes: for studies in which the model name is not explicitly specified in the manuscript, we consistently designate the model using the format “Author (Journal, Year)”, the same convention applies hereafter.

From a methodological standpoint, LLPS predictive modelling has followed a clear trajectory. Early studies predominantly relied on handcrafted physicochemical and sequence-derived features coupled with classical machine learning algorithms, such as support vector machines and random forests [132–139]. As larger datasets became available, deep learning architectures—including convolutional and recurrent neural networks [140–143]—were increasingly adopted to capture higher-order sequence patterns. More recently, Transformer-based protein language models have emerged as powerful representation learners, often used as embedding backbones combined with lightweight classifiers [144–148]. Many recent studies further adopt hybrid frameworks that integrate deep representation learning with traditional machine learning classifiers, reflecting a pragmatic response to limited training data and label noise (Table 3). For the purpose of the methodological taxonomy used in Table 3, the “Deep Learning” category is defined broadly to include both conventional neural-network-based architectures and transformer-based protein language/foundation model architectures.

**Table 3:** Categorization of LLPS prediction models by methodological framework and task coverage.

**Technology scope**	**Algorithm**	**Models**	**Task type**
Machine Learning	Random Forest	FLFB [86], catGRANULE 2.0 ROBOT [103], PSMutPred [124], dSCOPE [116], DeePhase [104], ML_LM [105], PSPHunter [92], PSAP [93], PSTP [101], Seq2Phase [128], PhaseNet [129], Ka Yin Chin *et al.* (BMC Bioinformatics, 2024) [106], Ashwin Lahorkar *et al.* (IEEE/ACM Transactions on Computational Biology and Bioinformatics, 2022) [109], Qinglan Ma *et al.* (Life (Basel), 2023) [111], Zahoor Ahmed *et al.* (Proteomics, 2024) [130]	I / II / III / IV
	XGBoost / CatBoost / GBDT / AdaBoost	Opt_PredLLPS [102], PSPire [88], MolPhase [89], PICNIC [91], PSPredictor [94], PhaSePred [127], Seq2Phase [128], PhaseNet [129], Ka Yin Chin *et al.* (BMC Bioinformatics, 2024) [106], Qinglan Ma *et al.* (Life (Basel), 2023) [111]	I / IV
	Logistic Regression	PULPS [126], PSMutPred [124], PSTP [101], Seq2Phase [128], FuzDrop [95], Ka Yin Chin *et al.* (BMC Bioinformatics, 2024) [106]	I / III / IV
	SVM / SVR	PSMutPred [124], Seq2Phase [128], Ka Yin Chin *et al.* (BMC Bioinformatics, 2024) [106], Ashwin Lahorkar *et al.* (IEEE/ACM Transactions on Computational Biology and Bioinformatics, 2022) [109]	I / III / IV
	HMM	PSPer [87], PLAAC [118]	I / II
	Stacked Ensemble	IDR-Puncta ML model [121]	II
Deep Learning	MLP	catGRANULE 2.0 ROBOT [103], Seq2Phase [128], PhosLLPS [125], PhaseNet [129], Soren von Bulow *et al.* (PNAS, 2025) [107], Wesley W. Oliver *et al.* (The Journal of Physical Chemistry B, 2025) [108], Zahoor Ahmed *et al.* (Briefings in Bioinformatics, 2025) [131]	I / III/ IV
	Attention/Transformer / Mamba	IFF [119], Droppler [97], ML_LM [105], PSTP [101], MambaPhase [98], PhaSeMotif [122], PhaseNet [129], Wenbin Li *et al.* (Briefings in Bioinformatics, 2025) [114], Yumeng Zhang *et al.* (Elife, 2025) [123]	I / II/ IV
	GNN/KNN	TIDGN [120], PhosLLPS [125], Ka Yin Chin *et al.* (BMC Bioinformatics, 2024) [106]	I / II/ III
	CNN	Opt_PredLLPS [102], PredLLPS_PSSM [100], PhaSeMotif [122], Zahoor Ahmed *et al.* (International Journal of Biological Macromolecules, 2024) [142]	I / II
	RNN / LSTM / GRU	Opt_PredLLPS [102], Droppler [97], PredLLPS_PSSM [100]	I
	Contrastive Learning/ Siamese Network	IFF [119], MambaPhase [98], snLLPS [99]	I / II
Others	Rule-based / Heuristic	ParSe [88], ParSe 2.0 [117], Jiyan Wang *et al.* (International Journal of Biological Macromolecules, 2023) [113]	I / II
	Monte Carlo / GA / SBO	catGRANULE [85], GP-GNN [96], LLPhyScore [79], Pscore [90], Pratik Mullick *et al.* (Biomolecules, 2022) [110]	I

Data dependency constitutes a central constraint shaping the current modelling landscape. Across the surveyed literature, training and evaluation datasets repeatedly draw from a small number of widely used sources, leading to substantial dataset reuse across studies. In practice, the datasets can be grouped into three broad origins: self-constructed datasets, publicly released LLPS-related databases and “other” auxiliary datasets; their usage patterns are consistent across Fig. 4c–e. Importantly, a portion of the “other datasets” are themselves inherited from earlier model-development efforts—e.g. PICNIC [91], PSTP [101], Opt_PredLLPS [102], PhaSeMotif [122], and PhaseNet [129] adopt the dataset released with PhaSePred [127] for training and/or testing—creating a cascade of dataset reuse that can propagate upstream curation choices and reduce independence across evaluations. Evaluation protocols also vary considerably, ranging from random splits to more stringent sequence-similarity-aware partitioning, which complicates direct comparison of reported performances. Together, these data-related factors influence not only model accuracy but also generalizability and reproducibility, underscoring the tight coupling between data resource design and algorithmic development in LLPS research.

### Task I: Liquid–liquid phase separation protein identification

The identification of phase-separating proteins represents the earliest and most extensively explored task in computational LLPS research [85, 86]. Given a protein sequence, this task aims to predict whether the protein has the intrinsic potential to undergo LLPS [88, 90]. Historically, this formulation has dominated the field, not necessarily because it best captures the biological nature of LLPS, but because it aligns well with available data and modelling conventions [96, 98, 99]. Early LLPS annotations were predominantly protein-centric, and the resulting labels could be readily cast as a binary classification problem, making this task particularly amenable to computational treatment [100–105]. As a consequence, the majority of existing LLPS predictors focus on protein-level propensity estimation [107, 108].

Despite the large number of published models, most approaches for LLPS protein identification can be broadly grouped into a small number of recurring modelling strategies. One prominent line of work is grounded in explicit biophysical assumptions, leveraging features related to intrinsic disorder, low-complexity regions, hydrophobicity, and specific intermolecular interactions such as π–π stacking. Representative models in this category, including LLPhyScore [79], MolPhase [89], and FuzDrop [95], explicitly encode physicochemical principles believed to stabilize biomolecular condensates. These methods often offer good interpretability and perform well for proteins conforming to known LLPS mechanisms, but their reliance on predefined interaction rules may limit generalizability to newly emerging or atypical phase separation behaviors.

A second major strategy adopts a more data-driven perspective, focusing on sequence-derived statistical patterns with minimal prior assumptions. Models such as PSPredictor [94], PICNIC [91], PSAP [93], and PSPHunter [92] exploit amino acid composition, residue-level features or sequence embeddings to discriminate LLPS proteins from non-phase-separating counterparts. In practice, many of these predictors are implemented with classical machine-learning backbones, such as random forests [149], support vector machines [150], and gradient-boosting methods [151, 152], reflecting a pragmatic balance between performance and limited labelled data. By emphasizing pattern recognition over mechanistic interpretation, these approaches can capture subtle sequence signatures and achieve robust performance across diverse datasets [109, 110]. However, their predictive power is tightly coupled to training data composition, and they are susceptible to dataset bias and limited biological interpretability [111–113].

A third, more recent direction attempts to move beyond purely protein-centric representations by incorporating contextual information relevant to phase separation. Examples include predictors that explicitly model protein–RNA interactions, protein–protein interaction networks, or experimental conditions, such as PSPer [87], Droppler [97], and the RNAPSEC team’s model [106]. By introducing interaction partners or environmental variables into the modelling framework, these approaches begin to approximate LLPS as a conditional and multi-component process rather than an isolated protein property. Although still relatively few in number, such models highlight the limitations of simplified propensity-based predictions and point toward more biologically faithful formulations.

Advances in deep learning and protein language models have further enhanced LLPS protein identification by providing powerful sequence representations. Architectures based on convolutional, graph-based, or attention mechanisms, as well as Transformer-derived embeddings, have consistently improved prediction accuracy [153, 154]. Recent studies also increasingly combine deep representation learning with classical classifiers in hybrid pipelines, aiming to stabilize training under small, noisy datasets while retaining strong feature expressivity. A ProtT5-based predictor integrating protein language model embeddings with KmerConv and multi-head attention has also been reported for LLPS protein identification, with evaluation performed on image-filtered negative samples across multiple independent datasets [114]. Importantly, these developments have primarily strengthened feature representation rather than redefining the task itself. Even highly accurate models remain constrained by the protein-level abstraction, answering whether a protein “resembles” known LLPS proteins rather than predicting when, where and, with which partners phase separation occurs. This inherent limitation motivates the exploration of alternative task definitions and modelling paradigms, providing the rationale for the event-centric perspective introduced next.

### Task II: Liquid–liquid phase separation-driving regions and sequence determinants

While Task I focuses on identifying whether a protein has the potential to undergo LLPS, a more mechanistically oriented question concerns which specific sequence regions within a protein drive this behavior. Task II addresses this problem by aiming to localize LLPS-promoting segments and uncover sequence determinants that contribute to condensate formation. This task reflects an important conceptual shift: rather than treating LLPS as a global protein property, it recognizes phase separation as an emergent outcome of localized sequence features and interaction motifs [118, 123]. Motivated by the central role of low-complexity regions, IDRs and prion-like domains in driving or modulating LLPS, a small set of studies has specifically focused on predicting LLPS-driving segments at the region level; to date, nine dedicated predictors in this category have been reported.

Early approaches to identifying LLPS-driving regions were closely linked to established biochemical insights, particularly the enrichment of IDRs, low-complexity domains and prion-like sequences in phase-separating proteins. Methods such as ParSe [115, 117] and dSCOPE [116] employed sliding-window strategies combined with physicochemical descriptors to score local sequence propensity for phase separation. These approaches offered interpretable connections between sequence composition and LLPS behavior, highlighting the contributions of charge patterning, aromatic residues, and disorder-associated features.

Subsequent models adopted more data-driven strategies to capture higher-order sequence patterns beyond predefined feature sets. By leveraging residue-level embeddings and deep learning architectures, tools such as IFF [119] and related predictors learned discriminative representations of LLPS-driving segments directly from annotated data. Attention-based mechanisms further enabled the identification of long-range dependencies and cooperative effects between distant residues, which are difficult to encode using handcrafted features alone. More recent work has also begun to refine this task from broad region localization toward motif-level characterization. For instance, PhaSeMotif focuses on essential motifs within phase-separating IDRs, whereas protein language model-based analyses have suggested that LLPS-relevant determinants may also appear as conserved continuous motifs containing both sticker-like and spacer-like residues [122]. Together, these methods improved localization accuracy and expanded the repertoire of detectable LLPS-associated sequence motifs. In addition, transfer-learning-based frameworks such as TIDGN [120] have been proposed to predict interaction/binding sites in intrinsically disordered proteins, providing complementary region-level signals that can be relevant to LLPS-driving or partner-dependent segments.

Despite these advances, predicting LLPS-driving regions remains inherently challenging. Boundaries between driving and non-driving segments are often diffuse, context-dependent, and sensitive to experimental conditions. Moreover, the functional impact of a given region frequently depends on its interaction with other biomolecules and its conformational dynamics, factors that are not fully captured by sequence information alone. Importantly, region-level predictions naturally lead to questions about how local sequence alterations influence phase separation behavior. In a related direction, the IDR-Puncta ML model [121] considered the condensate-forming behavior of individual IDRs in cells, and subsequent proteome-wide analyses linked predicted condensate-forming IDRs to proteins enriched in RNA processing and splicing functions. This consideration provides a direct bridge to Task III, where computational models aim to assess the effects of specific mutations on LLPS propensity and condensate properties.

### Task III: Predicting mutation effects on liquid–liquid phase separation

Building upon region-level identification of LLPS-driving sequences, Task III focuses on predicting how specific sequence variations modulate phase separation behavior. Rather than asking whether a protein or region can promote LLPS, this task addresses a more mechanistic and functionally relevant question: how mutations alter condensate formation, stability, or material properties. This formulation is particularly important for linking LLPS to genotype–phenotype relationships and disease-associated variants. In practice, mutation-focused predictors typically consider single-point or multi-point substitutions within a protein sequence and aim to infer how such perturbations change LLPS ability and behavior; however, systematic modelling studies dedicated to mutation effects remain relatively scarce compared with protein-level identification tasks, despite their importance for pinpointing regulatory sequence features and elucidating molecular mechanisms.

Early observations revealed that point mutations affecting charge distribution, aromatic residues, or disorder-promoting motifs can profoundly influence phase separation propensity. Computational models developed for this task aim to quantify such effects by comparing wild-type and mutant sequences, often integrating sequence context and physicochemical perturbations. Representative approaches, such as PSMutPred [124], frame mutation impact prediction as a supervised learning problem, leveraging curated mutation datasets to estimate whether a given variant enhances, disrupts or qualitatively alters LLPS behavior. A further development in this area is the incorporation of post-translational regulation at the residue level. PhosLLPS [125] predicts phosphorylation sites that regulate LLPS by combining protein language model embeddings with a graph-based deep learning framework, while the accompanying PTMPhaSe resource curates experimentally supported PTM sites with regulatory effects on LLPS. This is particularly relevant because residue-level perturbations in LLPS are not limited to sequence substitutions, but can also arise from reversible modifications that promote or inhibit condensate formation. These models enable systematic screening of variants that may not abolish phase separation entirely but subtly shift condensate dynamics.

The predictive scope of mutation-focused models extends beyond binary outcomes. In some cases, mutations may convert scaffold-like proteins into client-like roles, alter interaction specificity or bias condensates toward aberrant material states. Consequently, mutation effect prediction provides a direct computational route to understanding how LLPS dysregulation contributes to disease mechanisms, particularly in neurodegenerative disorders and cancer, where pathogenic mutations often cluster within intrinsically disordered or low-complexity regions. In addition to dedicated mutation-effect predictors, some region-oriented frameworks, such as PSPHunter [92], provide residue-level signals that can be leveraged to interpret how local substitutions may perturb LLPS-relevant sequence determinants.

Despite their promise, mutation-based predictors face substantial challenges. Available training data remain sparse and heterogeneous, with experimental measurements varying widely in assay type and resolution. Moreover, mutation effects are frequently context-dependent, influenced by interaction partners, expression levels, and cellular conditions. These limitations highlight the need to integrate mutation-level predictions with higher-order information about molecular roles and interaction patterns. While Task III focuses on quantifying variant-induced effects on phase separation, Task IV extends the analysis by classifying LLPS types and functional roles, thereby providing a higher-level interpretative framework for these perturbations.

### Task IV: Liquid–liquid phase separation types, roles, and dependency

Beyond predicting LLPS propensity or mutation-induced perturbations, a more biologically informative task is to characterize how proteins participate in phase separation. Task IV addresses this challenge by aiming to classify LLPS types, functional roles, and dependency patterns, such as distinguishing scaffold and client proteins or identifying partner- and condition-dependent phase separation behaviors. This task moves closer to a systems-level understanding of condensate organization, bridging molecular properties with functional outcomes.

Several computational approaches have been proposed to infer LLPS roles based on sequence features, interaction patterns, or learned representations. Models such as Seq2Phase [128] and PhaSePred [127] classify proteins according to their functional involvement in condensates, exploiting differences in sequence composition, disorder content, and interaction propensity between scaffolds and clients. These methods provide a structured interpretation of LLPS participation, enabling predictions that extend beyond binary phase separation labels [126]. By explicitly modelling functional categories, role-based predictors offer greater explanatory power for interpreting experimental observations and variant effects. More recent studies have further expanded this task by incorporating dependency relationships and contextual variables. Some predictors aim to distinguish spontaneous LLPS from partner-dependent or condition-specific phase separation, recognizing that many proteins only form condensates in the presence of specific interaction partners or under particular environmental conditions [131]. A similar tendency toward finer categorization is reflected in PhaseNet [129], which adopts a dual-task framework in which LLPS proteins are first separated from non-LLPS proteins and then further classified into self-assembling and partner-dependent categories. By combining protein language model embeddings with sequence-derived features for the first stage and an ensemble-based classifier for the second, it provides a more explicit operationalization of dependency patterns within LLPS prediction. By integrating protein–protein or protein–RNA interactions, as well as experimental context, these approaches begin to model LLPS as a conditional process rather than an intrinsic protein attribute.

Despite their conceptual significance, role- and type-based predictors remain relatively scarce. This limitation primarily reflects the lack of comprehensive, standardized annotations for LLPS roles and dependency patterns [130]. Moreover, functional roles are often dynamic, varying across cellular states and molecular compositions, posing additional challenges for static sequence-based models. Nevertheless, Task IV represents a critical step toward biologically faithful LLPS modelling. By framing phase separation in terms of roles, interactions and dependencies, it bridges molecular-level prediction and event-level representation, enabling the cross-cutting modelling paradigms and integrative frameworks outlined below.

### Cross-cutting modelling paradigms: from feature engineering to foundation representations

Across the diverse prediction tasks discussed above, a unifying theme in LLPS computational research is the continuous evolution of modelling paradigms driven by advances in protein representation. Rather than changes in classifier architecture alone, it is the transformation of how protein sequences are encoded that has fundamentally reshaped LLPS prediction strategies [101]. This evolution reflects a broader shift from explicit, hypothesis-driven feature engineering toward implicit, data-driven representation learning. Consistent with this trend, Fig. 4f shows the feature representation strategies used in the surveyed studies by reporting the number of models adopting each type of feature descriptor.

Early LLPS predictors were largely built upon handcrafted features derived from physicochemical principles and sequence statistics. Amino acid composition, charge patterning, intrinsic disorder, low-complexity regions, and interaction-related descriptors such as π–π stacking propensity were explicitly encoded to capture known drivers of phase separation [90]. These representations offered strong interpretability and were well aligned with prevailing biophysical models of LLPS. However, they also relied on predefined assumptions about sequence grammar and interaction mechanisms, limiting their ability to generalize across heterogeneous LLPS behaviors.

With the adoption of deep learning, representation learning emerged as a central component of LLPS modelling. Convolutional, recurrent, and attention-based architectures enabled models to learn task-specific embeddings directly from sequence data, reducing reliance on manual feature design [98]. More recently, the introduction of protein language models based on Transformer architectures has further decoupled representation learning from downstream prediction tasks. Pretrained on large-scale protein sequence corpora, these foundation models provide rich, context-aware embeddings that capture evolutionary, structural, and functional information [105]. In LLPS prediction, such embeddings are frequently combined with lightweight classifiers rather than end-to-end fine-tuning, reflecting both practical constraints and data characteristics.

Notably, hybrid modelling frameworks that integrate deep representations with classical machine learning classifiers have become particularly prevalent in LLPS research. This trend is not merely transitional but can be viewed as a rational response to current data limitations in the field. In many existing LLPS datasets, annotations remain relatively sparse, heterogeneous, and often noisy, with positive and negative labels defined under diverse experimental conditions. Under these dataset constraints, fully end-to-end deep models can be more prone to overfitting, whereas classical machine learning models benefit from robust optimization on limited datasets. Hybrid approaches leverage the expressive power of pretrained representations while maintaining stability and generalizability through simpler classifiers.

Despite substantial improvements in representation quality, an important mismatch persists between modelling capacity and task definition. Most LLPS predictors continue to operate under simplified, protein-centric formulations, using increasingly sophisticated embeddings to estimate intrinsic phase separation propensity. As a result, enhanced representations primarily improve discrimination within existing task boundaries rather than redefining what is being predicted. This limitation underscores that representation advances alone are insufficient to capture the conditional, multi-component and context-dependent nature of LLPS.

Finally, the evolution of modelling paradigms cannot be separated from concurrent advances in LLPS data resources. The growth of curated databases, expansion of sequence annotations and increasing integration of interaction and contextual information have directly enabled the adoption of foundation models and more expressive representations. Together, these trends highlight the co-evolution of LLPS data resources and algorithms, and motivate a shift toward event-level, multimodal modelling frameworks, as described below.

### Toward event-level and multimodal modelling of liquid–liquid phase separation

Despite substantial progress in LLPS prediction driven by improved representations and modelling strategies, a fundamental limitation persists across most existing approaches: the abstraction of phase separation as an intrinsic, protein-centric property. As discussed in previous sections, current predictive tasks—ranging from LLPS protein identification to region localization, mutation effect assessment and role classification—largely operate on isolated molecular entities. While these formulations have enabled methodological advances, they only partially reflect the biological reality of LLPS as a conditional, multi-component, and context-dependent process.

At the biological level, LLPS manifests as discrete events arising from cooperative interactions among multiple biomolecules, including proteins, nucleic acids and, in some cases, metabolites or polysaccharides, under specific physicochemical and cellular conditions [155]. Event outcomes are shaped not only by intrinsic sequence features but also by interaction partners, stoichiometry, environmental parameters, and spatiotemporal context [156]. Protein-centric predictors, even when powered by advanced representations, are inherently limited in their ability to capture such emergent behavior [157]. As a result, high predictive accuracy at the sequence level does not necessarily translate into mechanistic insight or functional interpretability.

These limitations point to the need for a paradigm shift from molecule-level prediction toward event-level modelling of LLPS. In this framework, the prediction target is no longer a single protein’s propensity to phase separate, but a structured LLPS event defined by participating components, interaction networks, contextual variables, and functional outcomes. Such a formulation aligns more closely with experimental observations and enables the integration of diverse data modalities, including protein–protein and protein–RNA interactions, expression dynamics, cellular localization, and environmental perturbations.

Recent advances in representation learning and foundation models provide a timely opportunity to support this transition [158]. Multimodal architectures capable of integrating sequence embeddings, interaction graphs, structural proxies, and contextual metadata offer a computational substrate for modelling LLPS events in a holistic manner [159]. Importantly, this shift necessitates corresponding changes in data resources, annotation standards, and evaluation protocols. Event-centric datasets with explicit representation of component composition and experimental context will be essential for training and validating next-generation models.

Ultimately, moving toward event-level and multimodal LLPS modelling represents more than a technical refinement; it reflects a conceptual realignment of computational objectives with biological complexity. By redefining prediction targets and embracing integrative data representations, future computational frameworks may not only improve predictive performance but also enable deeper mechanistic understanding of LLPS and its roles in physiology and disease.

## Event-centric frameworks for integrative liquid–liquid phase separation modelling and applications

### Redefining liquid–liquid phase separation as a computational event

Most existing computational studies conceptualize LLPS as an intrinsic property of individual biomolecules, predominantly proteins [81]. Under this molecule-centric paradigm, LLPS is formulated as a binary or probabilistic attribute inferred from sequence-derived features, with predictive efforts focused on identifying phase-separating proteins, their driving regions or mutation-sensitive residues [160]. While such formulations have enabled methodological advances and large-scale screening, they only partially capture the biological nature of phase separation.

From a biological perspective, LLPS does not arise from isolated molecular entities but emerges as a collective phenomenon driven by cooperative interactions among multiple components under specific conditions. Proteins, RNAs and, in some contexts, polysaccharides or metabolites jointly contribute to condensate formation, with outcomes shaped by stoichiometry, interaction networks, physicochemical environments, and spatiotemporal context [161–163]. The same molecule may participate in distinct phase separation behaviors depending on its partners, expression level or cellular state [164, 165]. Consequently, treating LLPS as a static, molecule-intrinsic attribute obscures the conditional and emergent properties that define phase separation *in vivo*.

This mismatch highlights a fundamental limitation in current computational formulations: prediction targets are misaligned with biological reality. Even highly expressive models and advanced sequence representations remain constrained when tasked with predicting an inherently context-dependent process using molecule-level abstractions [40]. Improved accuracy under simplified task definitions does not necessarily translate into mechanistic understanding, nor does it readily support functional interpretation or translational applications.

To address this gap, we propose that LLPS should be redefined, at the computational level, as a structured biological event rather than a molecular property. More specifically, an LLPS event may be defined as a context-bounded phase-separation process in which a particular set of molecular components, linked by interaction relationships and relative abundance constraints, gives rise under defined physicochemical and cellular conditions to an observable condensate state and associated functional consequences. In this framework, the term “event” does not merely denote the static existence of a condensate as an object; rather, it refers to a specific assembly–condition–behavior instance of phase separation. Accordingly, the minimal descriptive unit is not a single protein label but a component–condition–outcome unit that explicitly records who participates, how they are related, under what context phase separation occurs, and what material, functional or phenotypic outcome is observed [166]. This redefinition shifts the computational objective from estimating intrinsic propensities to modelling conditional behaviors arising from multi-component systems.

**Figure 5:**
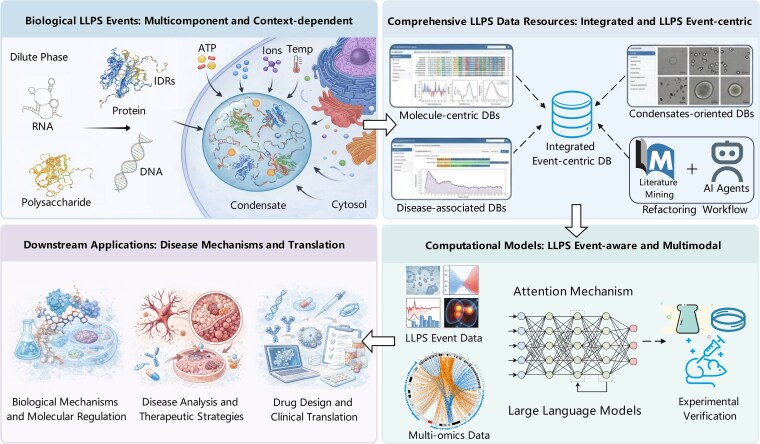
A roadmap for the future of LLPS research. The schematic outlines a four-module roadmap: (i) multicomponent, context-dependent LLPS events and their multi-molecular regulation; (ii) comprehensive, event-centric LLPS data resources that integrate molecule-centric, disease-associated, and condensate-focused databases via text mining and AI-agent-assisted curation; (iii) event-aware, multimodal computational models that combine integrated LLPS event data with multi-omics and large language model-enabled representations, supported by experimental validation; and (iv) downstream applications in biological mechanisms and molecular regulation, disease analysis and therapeutic strategies, and drug design and clinical translation.

This definition is consistent with broader views of biomolecular condensates as multicomponent, non-stoichiometric, and condition-sensitive assemblies whose composition, material properties, and functions vary across spatial and temporal scales [11, 167]. From a computational perspective, it also provides a tractable abstraction for integrating heterogeneous evidence and formulating predictive tasks that move beyond intrinsic propensity toward conditional, event-level reasoning. Figure 5 outlines key directions enabled by this event-centric perspective, spanning data representation, modelling paradigms and downstream applications. Importantly, such a perspective does not discard molecule-level predictions but embeds them within a higher-order framework that more faithfully reflects the biology of phase separation. By redefining LLPS as a computational event, subsequent data representation strategies, modelling approaches and application scenarios can be coherently aligned with biological complexity, providing a principled foundation for integrative and multimodal LLPS research.

### Event-level data representation: integrating multi-molecular and contextual dimensions

Redefining LLPS as a computational event necessitates a corresponding rethinking of data representation strategies. Current LLPS-related data resources are largely organized around individual entities, such as proteins or RNAs, with annotations describing intrinsic properties, sequence features, or experimental evidence of phase separation [168]. While such entity-centric representations have supported molecule-level prediction tasks, they are insufficient for modelling LLPS as a conditional and multi-component process. In an event-centric perspective, the fundamental data unit is no longer a single biomolecule but a structured LLPS event arising from specific combinations of components and contexts. Capturing such events requires data representations that explicitly encode not only what molecules are involved, but also how, under which conditions and with what functional consequences they interact. This shift transforms LLPS data from flat collections of annotated entities into relational and contextualized structures.

An event-level LLPS representation can be conceptualized as a structured schema comprising several interdependent dimensions. First, components define the participating biomolecules, including proteins, RNAs and, where relevant, polysaccharides or metabolites, together with their stoichiometric relationships. Second, interaction layers describe physical or functional associations, such as protein–protein or protein–RNA interactions, interaction strengths and network topology. Third, contextual variables capture experimental and cellular conditions, including *in vitro* versus *in vivo* settings, cellular compartment, stress states, and physicochemical parameters. Finally, outcomes characterize the observable properties of the event, such as condensate type, material state, dynamics, and functional relevance.

Importantly, such structured representations enable explicit modelling of conditionality and variability. The same molecule may participate in multiple LLPS events with distinct partners and outcomes, a reality that cannot be faithfully represented in molecule-centric databases. By contrast, event-level data structures naturally accommodate multiplicity, context dependence, and compositional diversity, aligning computational representations with biological observations. From an implementation perspective, event-level representations are well suited to relational data models and knowledge graph frameworks, where entities, interactions, and contexts can be integrated into a unified structure. This approach not only facilitates data integration across heterogeneous sources but also provides a foundation for downstream modelling strategies that explicitly reason over multi-component and multimodal information. As such, restructuring LLPS data around events is a critical prerequisite for advancing from descriptive resource compilation to truly integrative and mechanistic computational modelling.

### Event-aware and multimodal modelling strategies

The transition from molecule-centric to event-centric LLPS representations has profound implications for computational modelling strategies. When LLPS is framed as a structured event involving multiple components and contextual variables, traditional single-input predictors become inherently inadequate. Models designed to map individual sequences to scalar propensity scores are not equipped to reason over heterogeneous inputs, conditional dependencies, or emergent behaviors arising from component interactions [169]. Event-aware LLPS modelling requires algorithms capable of jointly integrating multiple information streams. At a minimum, such models must accommodate diverse molecular inputs, such as protein and RNA representations, relational information (interaction networks or co-participation patterns) and contextual descriptors (cellular state, experimental conditions, or perturbations). This shift transforms LLPS prediction from a single-task classification problem into a multi-input, multi-factor inference problem, where the outcome depends on coordinated patterns rather than isolated features.

Multimodal modelling frameworks offer a natural solution to these challenges. Architectures that combine sequence-based embeddings, interaction graphs, and contextual metadata enable the learning of representations that explicitly encode relationships among components. Graph-based models can capture interaction topology and dependency structure, while attention mechanisms facilitate flexible integration of heterogeneous modalities [170–173]. Importantly, multimodal fusion allows models to learn conditional rules [174], such as partner-dependent phase separation or context-specific condensate formation, which are difficult to express under molecule-centric formulations. Large pretrained models further expand the modelling toolkit for event-level LLPS analysis. Protein language models provide rich representations of individual components [146, 148, 175, 176], but their true potential emerges when they are embedded within higher-order frameworks that aggregate multiple component embeddings and contextual signals. In this setting, foundation models act as representation engines rather than end-to-end predictors, supplying semantically meaningful inputs to event-aware architectures. This design aligns well with current data realities, where limited event-level annotations favor modular and hybrid approaches over fully end-to-end training. Multi-omics profiles can further serve as complementary modalities for event-level LLPS modelling, including proteomics, transcriptomics, and genomics [177, 178]. Fused with sequence embeddings, interaction graphs and contextual metadata, these signals encode cellular state and perturbation context, enabling models to learn conditional rules such as cell-type-specific condensate formation and stress- or variant-dependent phase behaviors.

Crucially, event-aware modelling reframes predictive objectives. Instead of asking whether a molecule can phase separately, models aim to infer whether a specific combination of components under defined conditions gives rise to an LLPS event, and to characterize its properties. This perspective supports richer outputs, including probabilistic event occurrence, role assignments, and condition-dependent behavior. By aligning modelling strategies with event-level data structures, computational frameworks can move beyond improved discrimination toward mechanistic reasoning and hypothesis generation, laying the groundwork for meaningful biological and translational insights.

### Implications for disease mechanisms and translational applications

Reframing LLPS as an event-level phenomenon has important implications for understanding disease mechanisms and advancing translational applications. Many disease-associated perturbations linked to LLPS cannot be adequately explained by changes in the intrinsic properties of single molecules [179, 180]. Instead, pathological outcomes often arise from altered interactions, disrupted stoichiometry or context-dependent failures of condensate regulation [181]—features that are naturally captured at the level of LLPS events rather than isolated biomolecules.

In neurodegenerative disorders [182], e.g. disease-associated mutations frequently do not abolish phase separation per se but instead shift condensate material properties, alter partner preferences or promote aberrant maturation from liquid-like droplets to pathological aggregates [183, 184]. Event-level modelling enables these effects to be interpreted as transitions between distinct LLPS states driven by specific combinations of molecular components and conditions. By explicitly representing interaction context and event outcomes, computational frameworks can distinguish between benign, functional condensates, and disease-associated aberrant assemblies, providing a more nuanced mechanistic interpretation than molecule-centric predictors. Similarly, in cancer and other complex diseases, LLPS often acts as a regulatory layer that modulates signaling, transcriptional control, and stress adaptation [185, 186]. Perturbations in expression levels, post-translational modifications or interaction networks can rewire phase separation events without fundamentally altering individual protein sequences. Event-centric representations allow such regulatory rewiring to be modelled as shifts in event composition and context, offering a systems-level perspective on how LLPS contributes to disease progression and cellular heterogeneity.

From a translational standpoint, event-level modelling also reshapes strategies for therapeutic intervention. Traditional drug discovery paradigms focus on targeting individual proteins or disrupting specific binding sites [83]. However, LLPS-mediated functions frequently depend on collective behaviors and mesoscale material properties [187, 188]. Event-aware frameworks enable the exploration of intervention strategies aimed at modulating event composition, interaction strength, or environmental conditions, rather than simply inhibiting single molecular targets. This perspective aligns with emerging efforts to regulate condensate dynamics, stability, or reversibility as therapeutic endpoints. Beyond disease, event-centric LLPS modelling has implications for synthetic biology and biomolecular engineering. Designing synthetic condensates or programmable phase behaviors requires predicting outcomes of multi-component systems under defined conditions—precisely the type of problem addressed by event-level representations and multimodal models. By providing a unified framework that links molecular components, context and emergent properties, event-based approaches facilitate rational design and functional control of phase-separated systems.

Together, these considerations highlight that treating LLPS as a computational event is not merely a conceptual refinement, but a practical necessity for translating computational insights into mechanistic understanding and real-world applications. By aligning data, models and biological questions around a shared event unit, this perspective makes it easier to trace explicit component–condition–outcome relationships and to perform comparable inference across cellular states and disease contexts. Moreover, event-centric representations naturally support actionable outputs—such as state transitions, role assignments, and context-dependent intervention points—thereby narrowing the gap between computational prediction, experimentally testable hypotheses, and translational strategies.

## Challenges and outlook: from event modelling to translational liquid–liquid phase separation biology

Despite the conceptual and practical advances achieved in LLPS data curation and predictive modelling, several fundamental challenges continue to limit the mechanistic interpretability and translational impact of current computational approaches. At the core of these challenges lies the intrinsic nature of LLPS as a multicomponent, condition-dependent phenomenon that emerges from collective molecular behavior rather than from isolated biomolecules. Although molecule-centric abstractions have enabled scalable dataset construction and efficient model training, they remain poorly aligned with the biological reality of phase separation, particularly when contextual modulation and conditional outcomes are central to function.

A primary bottleneck for advancing beyond molecule-centric frameworks is the limited availability of high-quality, event-level annotations. Most existing LLPS datasets remain organized around individual proteins or nucleic acids, with heterogeneous definitions of phase separation, inconsistent experimental conditions and incomplete contextual metadata. In many cases, records lack a consistent schema specifying which molecular components co-participate, under what physicochemical or cellular regimes, and with which observable outcomes—precisely the information required to treat LLPS as a structured computational event. Addressing this limitation will require coordinated community efforts in data curation, reporting standards and annotation practices, including shared vocabularies, explicit evidence codes, and transparent provenance tracking. Minimal reporting requirements for component stoichiometry, perturbations, and assay readouts would further enable event definitions that are interoperable and machine-actionable.

A second major challenge arises at the modelling level. Event-centric representations necessarily introduce high-dimensional and heterogeneous inputs that encompass multiple biomolecules, interaction networks, and contextual variables. While multimodal and graph-based learning architectures provide powerful tools for such integration, they also impose increased demands on data volume, computational resources, and interpretability [189]. Moreover, event-level data will inevitably be incomplete: interaction graphs may be partial, contextual descriptors missing, and outcome labels coarse or assay-dependent. Robust modelling therefore requires principled strategies for handling uncertainty and missing modalities, such as modular architectures that combine pretrained encoders with event-level aggregation layers, weakly or semi-supervised learning, and interpretability methods that attribute predictions to specific components, interaction motifs, or contextual factors rather than opaque latent features.

Validation constitutes a further bottleneck that becomes particularly pronounced at the event level. Unlike molecule-centric predictions, which can often be benchmarked against curated positive and negative sets, event-level predictions demand context-aware experimental validation. This requirement complicates performance assessment and underscores the necessity of close integration between computational modelling and experimental design. Prospective validation may involve targeted reconstitution assays, cellular perturbation experiments or controlled manipulation of component stoichiometry and environmental parameters to test predicted conditional behaviors and state transitions [190]. In this setting, iterative feedback loops between prediction and validation are essential, and active-learning–style strategies that prioritize experiments with maximal information gain may help focus experimental effort where it is most informative.

Despite these challenges, the outlook for event-level LLPS research remains highly promising. Advances in high-throughput experimental technologies, improved reporting of interaction and condition metadata, and the continued expansion of curated LLPS resources are gradually enriching the available data landscape. In particular, emerging multi-omics datasets, spatially resolved measurements and systematic perturbation screens are beginning to provide the contextual richness required to parameterize LLPS beyond single-molecule labels [191]. In parallel, rapid progress in foundation models and multimodal learning architectures offers an increasingly powerful computational substrate for modelling complex biological events. Together, these developments suggest that many of the technical prerequisites for event-aware LLPS modelling are now coming into place. However, the availability of richer data and more powerful models does not, by itself, guarantee a corresponding shift in conceptual abstraction or analytical framing.

Nevertheless, current progress in LLPS bioinformatics remains largely incremental at the level of data abstraction and task formulation. Although expanding data resources and increasingly powerful learning architectures have improved coverage and predictive capacity, most existing datasets and models are still organized around individual molecules or simplified propensity labels [192]. At the same time, recent predictive studies have begun to move beyond coarse-grained protein-level identification toward more mechanistically informative questions, including the delineation of LLPS-driving regions, the assessment of mutation or post-translational modification effects, and the classification of dependency patterns or functional roles. This shift suggests a growing effort to extract deeper biological meaning from LLPS prediction, even though most current formulations remain grounded in molecule-centric abstractions. As a result, multicomponent assembly logic, environmental dependence, and context-specific phase behaviors remain insufficiently represented in current computational frameworks. This continuing abstraction gap reinforces our central argument that next-generation LLPS bioinformatics should move beyond molecule-centric formulations toward explicitly event-centric frameworks capable of encoding assembly–condition–behavior relationships and supporting integrative, context-aware modelling.

Taken together, these observations reinforce a central conclusion of this review: continued refinement of molecule-centric predictors alone is unlikely to resolve the core conceptual and practical challenges of LLPS modelling. Instead, progress will increasingly depend on a decisive shift toward event-centric frameworks that explicitly encode molecular assemblies, contextual conditions, and observable phase behaviors. Treating LLPS as a structured computational event offers a unifying abstraction that can bridge molecular features, interaction networks, and cellular context, align computational outputs with experimental observables, and support more interpretable and actionable predictions. Such a perspective not only advances fundamental understanding of phase separation but also provides a stronger foundation for disease mechanism analysis and translational applications, where conditional and context-specific behaviors are paramount. Ultimately, broad adoption of event-centric LLPS modelling will require sustained community alignment around shared definitions, transparent evidence hierarchies, and interoperable standards, enabling integrative and reproducible analysis to become routine rather than bespoke.

## Conclusion

LLPS has emerged as a fundamental principle underlying cellular organization, regulation, and disease. In parallel with rapid experimental advances, bioinformatics and computational biology have become indispensable for extracting patterns, formulating hypotheses, and enabling large-scale analysis of LLPS-related data. In this review, we systematically examined current bioinformatics resources and predictive models for LLPS, providing a comprehensive synthesis of available databases and computational tasks while highlighting both major methodological advances and persistent conceptual limitations.

Our analysis demonstrates that, despite increasingly sophisticated representations and modelling techniques, most existing computational studies remain anchored to molecule-centric formulations that treat LLPS as an intrinsic property of individual biomolecules. While such abstractions have enabled scalable data integration and efficient predictor development, they only partially capture the conditional, multicomponent, and context-dependent nature of phase separation. As a result, improvements in predictive performance do not always translate into deeper mechanistic insight or translational relevance. To address this disconnect, we proposed a unifying event-centric perspective that reframes LLPS as a structured biological event defined by participating components, interaction relationships, contextual conditions, and functional outcomes. Building on this definition, we outlined how data resources, modelling strategies, and application scenarios can be coherently reorganized around LLPS events.

Within this framework, next-generation bioinformatics in LLPS should treat the event—rather than the isolated molecule—as the primary unit of representation, curation, and prediction. On the resource side, this entails the development of event-level datasets that explicitly encode environmental dependence, including temperature, pH, ionic strength, macromolecular crowding, subcellular localization, and stress states; molecular dependence, spanning partner identity, stoichiometry, competition or cooperativity, post-translational modification, and mutation effects; and dynamic evolution, encompassing assembly and disassembly kinetics, material-state transitions, maturation trajectories, and aberrant aggregation. On the modelling side, predictor development should likewise proceed from an event-based formulation, leveraging multimodal architectures that integrate sequence and structure representations with interaction graphs and contextual metadata, and further incorporating AI-enabled foundation models together with multi-omics signals from proteomic, transcriptomic, epigenomic, and spatially resolved measurements to infer conditional behaviors and emergent properties. Importantly, such an approach provides a more faithful bridge between computational prediction and biological interpretation, particularly in the study of disease mechanisms and translational applications.

Looking ahead, the realization of next-generation LLPS bioinformatics will require coordinated progress in data standardization, event-level annotation, and integrative modelling. By aligning computational abstractions with biological complexity, event-centric frameworks have the potential to shift LLPS bioinformatics from descriptive prediction toward mechanistic understanding and rational intervention. We anticipate that this paradigm will play an increasingly central role in shaping future LLPS research across basic, biomedical, and applied domains.

**Key Points**

- LLPS is a biological event, not a molecule-level trait—it is inherently multicomponent and condition-dependent.
- The current LLPS bioinformatics ecosystem remains fragmented and predominantly molecule-centric: resources vary in evidence standards and annotation consistency, and existing predictive models often underrepresent event-level context and mechanistic structure.
- Next-generation bioinformatics in LLPS: make events the unit of curation and modelling—standardize evidence, annotate event states, and build integrative models that advance from prediction to mechanism to intervention.
- For data resources, a central priority is to move from molecule lists to interoperable event graphs that explicitly link components/stoichiometry, physicochemical conditions, spatiotemporal dynamics and material outcomes, supported by unified standards, evidence tiers, and clear provenance.
- In predictive modelling, move beyond propensity scores to event-aware multimodal AI integrating sequence/structure, interaction networks and context, augmented by foundation-model representations and multi-omics to learn conditional rules and emergent behaviors.

## Supplementary Material

Supplementary_material_bbag254

## Data Availability

This study does not produce or analyze new data.
