# Supplementary material for: Rethinking bioinformatics in liquid–liquid phase separation: data resources, predictive models, and an event-centric perspective
Source: Brief Bioinform. 2026 May 25;27(3):bbag254. doi: 10.1093/bib/bbag254 (PMC13200548; doi:10.1093/bib/bbag254)
Supplement: Supplementary_material_bbag254 [file supplementary_material_bbag254.zip › Table S1.docx]

Table S1. Search Formula for LLPS Prediction Models (PubMed as an Example)

| Search Formula | (("liquid-liquid phase separation"[All Fields] OR "phase separation"[All Fields] OR "LLPS"[All Fields] OR "biomolecular condensate"[All Fields] OR "protein condensates"[All Fields] OR "membraneless organelle"[All Fields] OR "intrinsically disordered protein"[All Fields] OR "intrinsically disordered regions"[All Fields] OR "IDP"[All Fields] OR "low complexity region"[All Fields] OR "scaffold proteins"[All Fields] OR "regulator proteins"[All Fields] OR "RNA-binding proteins"[All Fields] OR "aggregates"[All Fields] OR "supersaturation"[All Fields] OR "sequence segments"[All Fields] OR "phase separating proteins"[All Fields] OR "phase-separating proteins"[All Fields] OR "protein phase separation"[All Fields] OR "protein biophysics"[All Fields] OR "protein phase transition"[All Fields] OR "protein self-assembly"[All Fields] OR "subcellular organelle"[All Fields] OR "phase separation RNA"[All Fields] OR "protein droplets"[All Fields]) AND ("prediction"[All Fields] OR "predictor"[All Fields] OR "machine learning"[MeSH Terms] OR "deep learning"[All Fields] OR "artificial intelligence"[MeSH Terms] OR "computational model"[All Fields] OR "algorithm"[All Fields] OR "in silico"[All Fields] OR "neural network"[MeSH Terms] OR "random forest"[All Fields] OR "support vector machine"[All Fields] OR "natural language processing"[All Fields] OR "NLP"[All Fields] OR "AlphaFold"[All Fields] OR "protein language model"[All Fields] OR "pre-trained protein language model"[All Fields] OR "convolutional neural network"[All Fields] OR "classification model"[All Fields] OR "graph neural networks"[All Fields] OR "LLMs"[All Fields] OR "large language models"[All Fields] OR "language model"[All Fields] OR "model training"[All Fields] OR "protein sequence analysis"[All Fields] OR "molecular simulations"[All Fields] OR "feature fusion"[All Fields])) |
| --- | --- |
