# Supplementary material for: Rethinking bioinformatics in liquid–liquid phase separation: data resources, predictive models, and an event-centric perspective
Source: Brief Bioinform. 2026 May 25;27(3):bbag254. doi: 10.1093/bib/bbag254 (PMC13200548; doi:10.1093/bib/bbag254)
Supplement: Supplementary_material_bbag254 [file supplementary_material_bbag254.zip › Figure S1 caption.docx]

Figure S1: Article search strategy (inclusion and exclusion criteria). This figure illustrates the literature inclusion and exclusion workflow for studies on phase-separation–related data resources and predictive models included in this review, the screening process was conducted in accordance with the PRISMA guidelines."
